# Using Scientific Articles on COVID-19 to Introduce the Nature of Scientific Knowledge to Medical Students

**DOI:** 10.1007/s40670-023-01874-0

**Published:** 2023-08-24

**Authors:** Georgios Ampatzidis, Anastasia Armeni

**Affiliations:** 1 Department of Early Childhood Education, University of Thessaly, Argonafton & Filellinon, 38221 Volos, Greece; 2 Department of Endocrinology, School of Medicine, University of Patras, 26504 Rio-Patras, Greece

**Keywords:** Nature of scientific knowledge, COVID-19, Medical students, SARS-CoV-2

## Abstract

It has been argued that, as evidence-based medicine emerged in the 1990s, healthcare practitioners are required to partake in more intricate and elaborate cognitive processes. As a result, knowing the characteristics and constraints of scientific knowledge — i.e., an advanced understanding of the Nature of Scientific Knowledge (NOSK) — has become progressively imperative. In this paper, we discuss snapshots of the research on SARS-CoV-2 that may be used in order to illustrate aspects of NOSK to medical students and how they may be introduced within teaching interventions.

## Introduction

The practice of medicine operates at the intersection of individual clinical proficiency, social values, and science. While all of these factors are important, it’s worth noting that knowledge from basic and clinical sciences, such as biochemistry, physiology, and pathology, plays a crucial role in shaping and informing clinical medicine. This scientific knowledge helps to explain and give context to clinical practices related to health, disease, and treatment. A set of epistemological, logical, and ethical principles form the basis of scientific knowledge [1]. The epistemological principles related to scientific knowledge are often referred as Nature of Scientific Knowledge (NOSK) which is considered a part of Nature of Science [2].

Although formulating a widely accepted definition for NOSK is challenging, it is considered to deal with broader philosophical traits of scientific content and methodologies that are likely to be applicable, albeit to varying extents, across various scientific disciplines [3]. The understanding of NOSK is considered to play an influential role that conditions the cognitive process [1]; the way we comprehend, assimilate, and utilize knowledge is influenced by our ideas regarding NOSK [4]. Moreover, a significant understanding of NOSK has been linked with advanced critical thinking skills [5]. It has been argued that, as evidence-based medicine emerged in the 1990s, healthcare practitioners are required to partake in more intricate and elaborate cognitive processes. Thus, knowing the characteristics and constraints of scientific knowledge and methodology — i.e., an advanced understanding of NOSK — has become progressively imperative [4].

Despite its significance for students, understanding and teaching NOSK appears to be challenging [6]. In an investigation with medical students, Köksal and Köksal reported that their informants revealed many misunderstandings concerning aspects of NOSK [7]. The authors concluded that their participants lacked adequate comprehension to resolve issues that required informed decision-making. Pettersen drew on the results of surveying health sciences students in regards to their understanding on NOSK to argue on the relevance of teaching about NOSK to students of this background [8]. Peña, Paco, and Peralta also suggested that promoting NOSK understanding is crucial to enhance the implementation, education, and acquisition of medical practice [1].

A popular approach to NOSK teaching and learning is through a set of general aspects, for instance, through aspects like the tentativeness of scientific knowledge or the creativity involved in scientific knowledge [9]. The general aspects of NOSK are usually introduced to students in a contextual manner, which implies that they are linked to specific scientific content. This integration may take place in various forms, such as student-led laboratory investigations [10], lessons centered on the history of science [11], or lessons centered on ongoing scientific research [12].

Considering the above, in this article, we suggest a way to introduce NOSK to medical students through using information on the research conducted on SARS-CoV-2. More specifically, we (a) consider snapshots of the research on SARS-CoV-2 discussed in scientific articles that may be used in order to illustrate aspects of NOSK, and (b) discuss their classroom use. The aspects of NOSK that concern us here are the following: (a) scientific knowledge involves creativity, (b) scientific knowledge is tentative, and (c) scientific knowledge is socio-culturally embedded.

## Scientific Knowledge Involves Creativity

Several approaches have been tested in order to develop a treatment for SARS-CoV-2. From the onset of the COVID-19 pandemic, researchers around the world have been searching for analogies between SARS-CoV-2 and known pathogens to administer medicines used for other diseases as a therapy for COVID-19. For instance, at the beginning, it was suggested that the bacillus Calmette-Guérin (BCG) vaccine, originally developed to prevent tuberculosis, may have wider protection against various infectious diseases and it was proposed that it may potentially mitigate the severity of COVID-19. Escobar, Molina-Cruz A, and Barillas-Mury reported that, adjusting for various confounding factors, significant associations were found between BCG vaccination and lower mortality rates from COVID-19 [13]. Different approaches tested included passive immunotherapy (e.g., convalescent serum and monoclonal antibodies), kinase inhibitors, adoptive immunotherapy, mesenchymal stromal cells, nanomedicine, decoy biomolecules, antiviral drugs, and corticosteroids [14].

On the other hand, several researchers were involved in developing a vaccine against COVID-19 as well. In a review published in April 2020, Le et al. mentioned that there were already 115 vaccine candidates, 78 of which were confirmed as active and 73 out of them were at that time currently at exploratory or preclinical stages [15]. As of July 2021, there were 184 COVID-19 vaccine candidates in the pre-clinical development stage, with 105 undergoing clinical development. Additionally, at least one regulatory authority had approved 18 vaccines for emergency use. These potential vaccines may be categorized based on the technology used to achieve immunity (i.e., protein-based vaccines, whole virus vaccines, nucleic acid vaccines, viral vector vaccines). Despite sharing some commonalities, each vaccine had unique features developed by implementing creative design and techniques [16].

## Scientific Knowledge Is Tentative

Since the emergence of COVID-19, a lot of research has been undertaken about the nature of SARS-CoV-2 and new data often subverted what scientists believed at preliminary research stages. For instance, at first, it was suggested that patients infected with SARS-CoV-2 may had visited the Wuhan seafood market or may had consumed infected animals sold there. However, soon it was revealed that several people contracted the virus without having visited the market, which suggested a human-to-human transmission [17]. Another example is scientists’ ideas about the systems SARS-CoV-2 infected. The first patients were diagnosed with viral pneumonia and the novel coronavirus was identified as a virus of the respiratory system. However, subsequent research data suggested that SARS-CoV-2 may infect other systems as well. For instance, the coronavirus seemed to have an affinity for the gastrointestinal system; biopsies and autopsy examinations using electron microscopes have shown that the virus replicates vigorously in intestinal tissue. The SARS-CoV-2 has been found to affect also the urogenital system, the central nervous system, and the circulatory system [18].

Finally, children’s role in spreading the virus has been in focus from the beginning of research on SARS-CoV-2. Preliminary research data suggested that although children were less likely to experience severe symptoms, they were still exposed to similar infection risks as the general population which highlighted the fact that targeting this group could still be crucial for interventions that aimed to minimize transmission, even if they did not get sick [19]. If children were the main carriers of household transmission of SARS-CoV-2, similar to what is observed in influenza, then the possibility of silent spread from infected children who were unaware of their condition could be a crucial contributor to the spread of the virus in the community. On this presumption, initially schools shut down due to concerns about widespread transmission among young students in many countries, but later on — in the light of a growing body of evidence demonstrating that children faced a lower risk of contracting coronavirus and spreading it to others — it appeared that schools could resume operations [20].

## Scientific Knowledge Is Socio-culturally Embedded

The COVID-19 pandemic has garnered worldwide attention due to its impact on global security and the economy. The pandemic has prompted researchers from all over the world to collaborate on an unprecedented scale and timeline to combat a single disease [21]. While researchers working in research fields related to SARS-CoV-2 reported no change or even an increase of their workload, many scientists reported a significant decrease in the average time spent on research during the first months of the pandemic — see, for instance, the Myers et al.’s report of an April 2020 survey among 4500 principal investigators [22].

Scientists have not been impacted by COVID-19 equally. Researchers in disciplines such as biochemistry, chemistry, and chemical engineering, which heavily rely on physical laboratories and time-sensitive experiments, reported the most significant reduction in research time. Nevertheless, fields such as mathematics, statistics, computer science, and economics, which are less equipment-intensive, reported the smallest declines in research time [23]. On the other hand, COVID-19 has become a significant area of research interest for numerous researchers around the world many of whom switched because the only way for them to reopen their labs was by studying the virus. According to Michael Lauer from the US National Institutes of Health, approximately 80% of clinical trials were either halted or disrupted. This disruption occurred because resources were redirected towards managing the pandemic, leading many researchers to pause their own studies in order to prioritize patient care in crowded hospitals. Moreover, long-planned trials concerning other infectious diseases were put on hold as researchers shifted their focus to studying COVID-19 [24].

## Classroom Use

Students may be introduced to the target NOSK aspects in special sessions in which they will receive a worksheet containing information on the snapshots of the SARS-CoV-2 research mentioned above. They will be required to collaborate in pairs to respond to questions based on the text aiming to guide them towards the desired conclusions about NOSK. At the end of the session, peer groups will share their responses with the class in a teacher-facilitated discussion. The use of open-ended questions in the worksheets is supposed to encourage students to reflect and engage in critical dialogue while reading in groups and writing down their thoughts. Examples of the questions that may be used are the following: “Which traits of scientists’ personalities should be activated in order (a) to enable them to envision the use of drugs originally intended for other diseases to treat SARS-CoV-2 and (b) to create numerous different vaccines for SARS-CoV-2? What does this imply for NOSK?’; “Why did scientists alter their initial beliefs regarding (a) the animal-to-human transmission of SARS-CoV-2, (b) the organs and systems infected by the virus, and (c) the transmission of SARS-CoV-2 by children? What does this imply for NOSK?”; “Ηow has the pandemic affected (a) the way that scientific research is conducted, and (b) the relevant scientific knowledge? What does this imply for NOSK?”.

## Final Remarks

Our argument in this paper is that text combining information from the research on SARS-CoV-2 may serve as an effective tool for explicitly teaching NOSK aspects to medical students. The creativity involved in scientific knowledge, the provisional nature, and the socio-scientific embeddedness of scientific knowledge may be effectively portrayed through lessons centered on current scientific research. The implementation of specific techniques, such as group reading and open-ended questions, further supports students in exploring these NOSK aspects when using this text as part of teaching interventions.

There are several teaching and learning strategies used in medical education which have been proved to be effective in medical classrooms. For instance, problem-based learning (PBL), case-based learning (CBL), and team-based learning (TBL) have long been implemented and tested with positive learning outcomes. We do not aim to propose a new teaching and learning strategy; we rather suggest the use of materials that we consider appropriate for introductory sessions on NOSK and discuss their classroom use. We consider what we discuss in this article as an engaging way to introduce NOSK to first-year medical students that could fit in the crowded curriculum of medical schools. Of course, educators may adapt our suggestion to their own teaching practices and use information on SARS-CoV-2 discussed in scientific articles as part of teaching interventions based on, for instance, TBL.

There are studies focusing on how to promote NOSK aspects through information on the COVID-19 research [25–27]. In these studies, aspects of the research on SARS-CoV-2 research are introduced to K-12 students and preservice teachers through news articles. In our paper, we argue on the use of information on SARS-CoV-2 communicated through scientific articles for teaching NOSK to medical students. Undoubtedly, gathering empirical evidence on the effectiveness of using information from the research on SARS-CoV-2 in a classroom setting is crucial. Therefore, subsequently we plan to implement a case study wherein medical students will collaborate in pairs to respond to questions based on information on COVID-19. Collecting data of their performance will help us evaluate whether and how such a teaching intervention can enhance students’ comprehension of the target NOSK aspects.

## Data Availability

N/A.

## Code Availability

N/A.
